# Supplementary material for: Drosophila STING protein has a role in lipid metabolism
Source: eLife. 2021 Sep 1;10:e67358. doi: 10.7554/eLife.67358 (PMC8443252; doi:10.7554/eLife.67358)
Supplement: Figure 1—figure supplement 1—source data 1. [file elife-67358-fig1-figsupp1-data1.pdf]

Source data for Supplementary Figure 1B

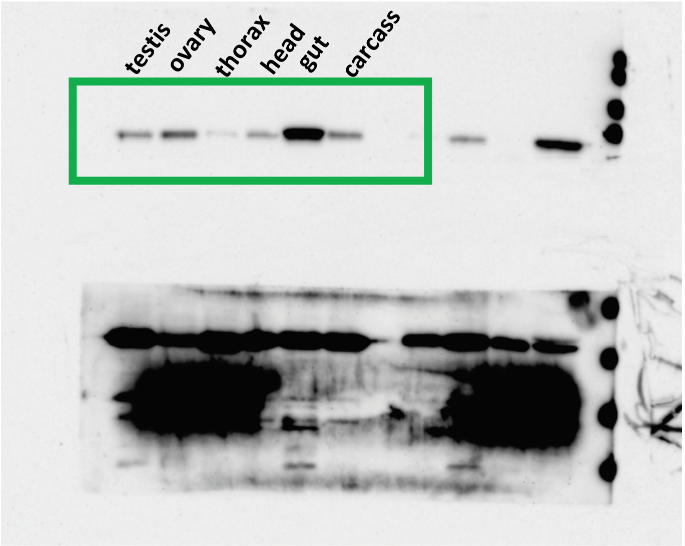

anti-GFP WB

Source data for Supplementary Figure 1D

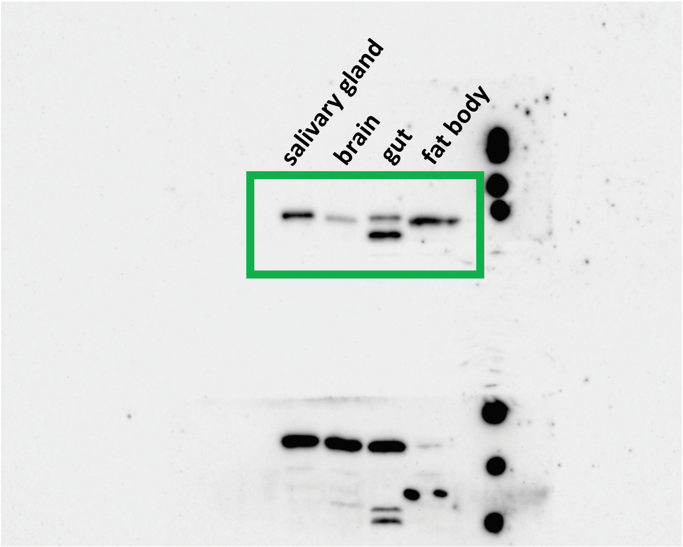

anti-GFP WB
