# Supplementary figures and images for: Drosophila STING protein has a role in lipid metabolism
Source: eLife. 2021 Sep 1;10:e67358. doi: 10.7554/eLife.67358 (PMC8443252; doi:10.7554/eLife.67358)

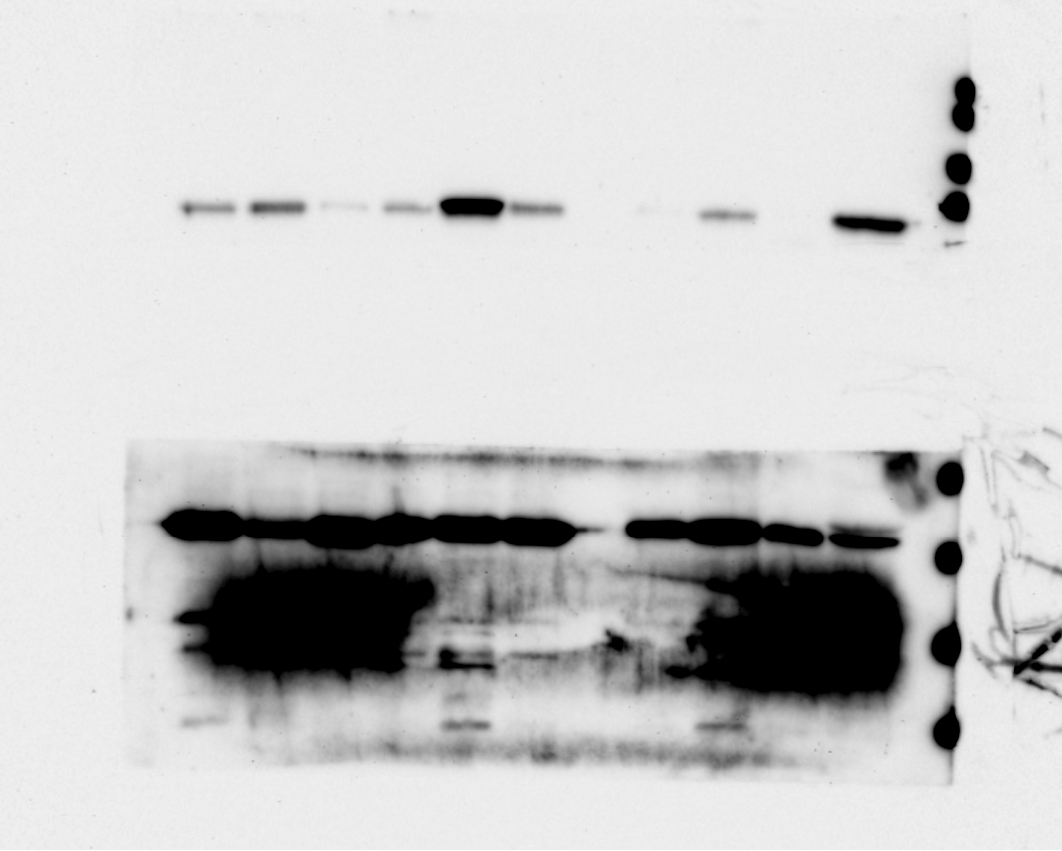

Supplement: Figure 1—figure supplement 1—source data 2. [file elife-67358-fig1-figsupp1-data2.tif]

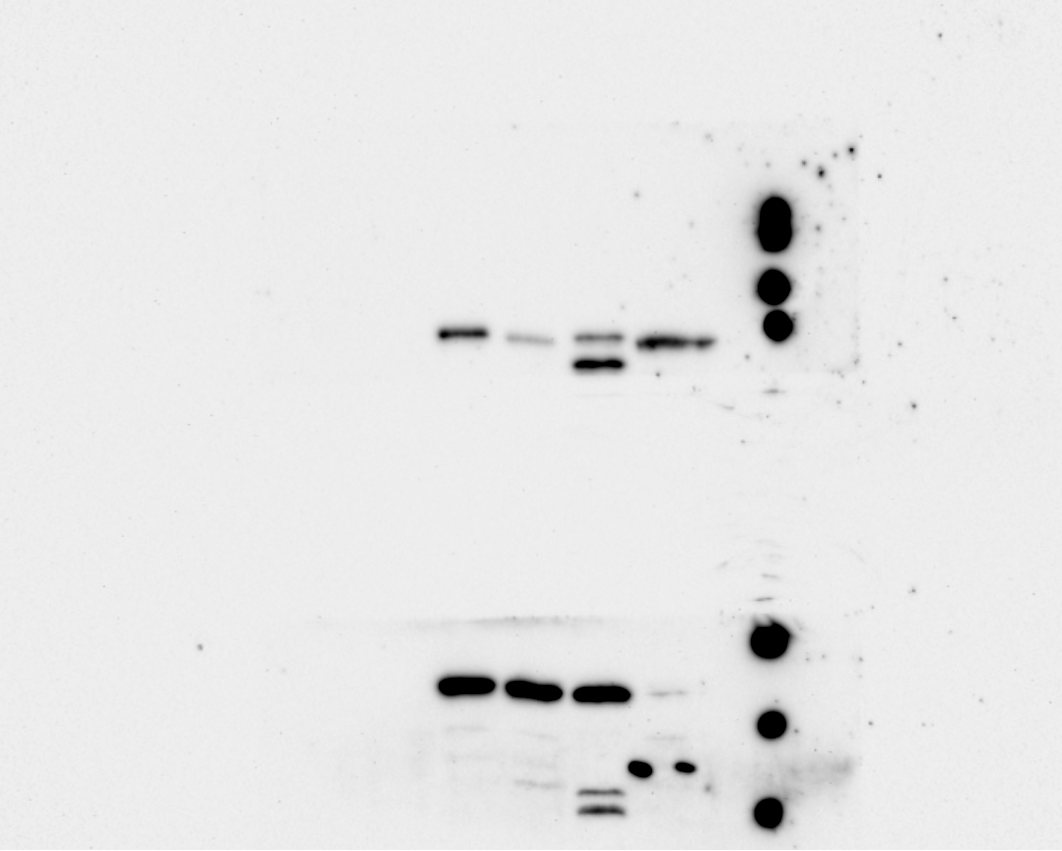

Supplement: Figure 1—figure supplement 1—source data 3. [file elife-67358-fig1-figsupp1-data3.tif]

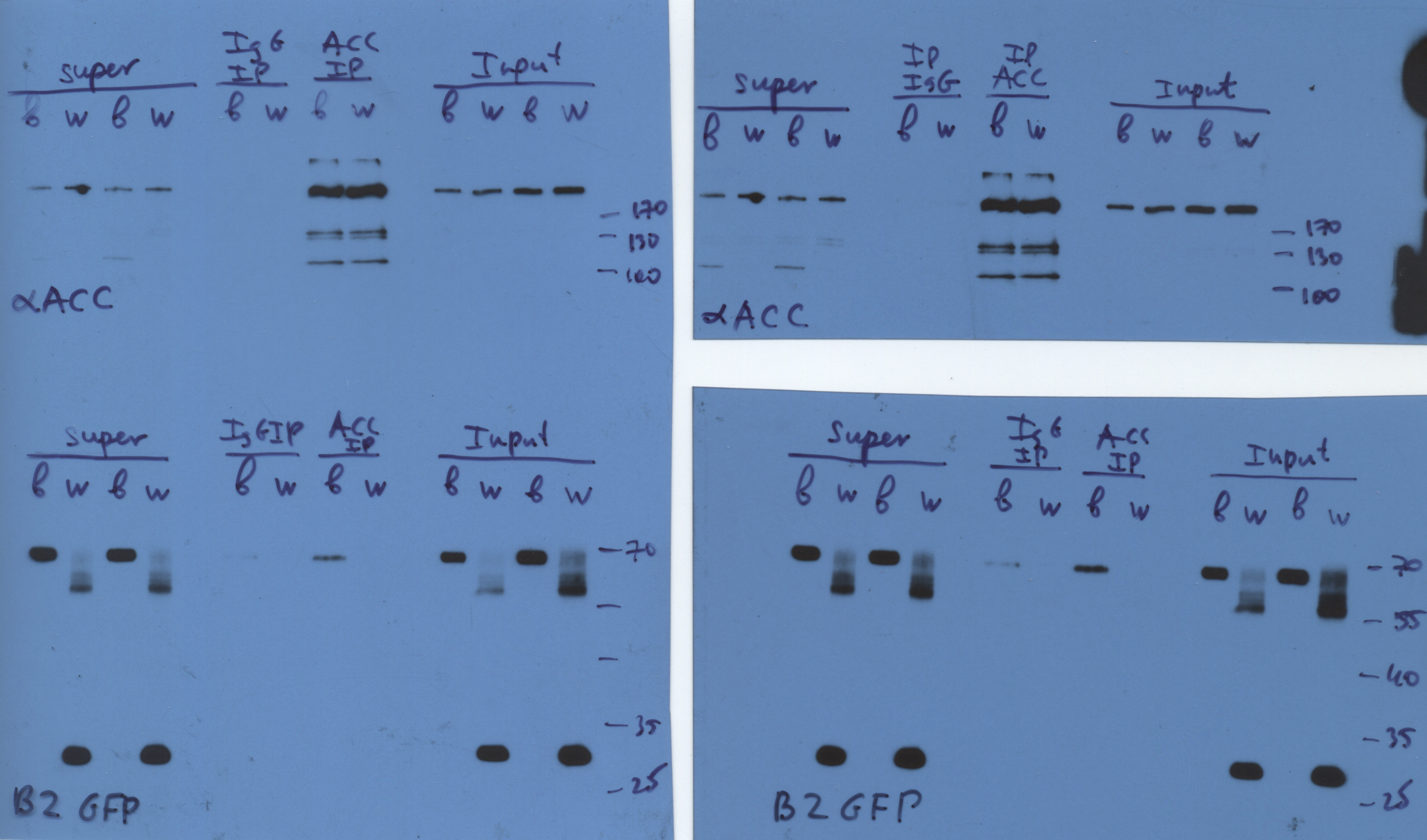

Supplement: Figure 4—source data 2. [file elife-67358-fig4-data2.zip › 31c2f395-8c08-49ed-a2b2-8a484039527a.jpeg]

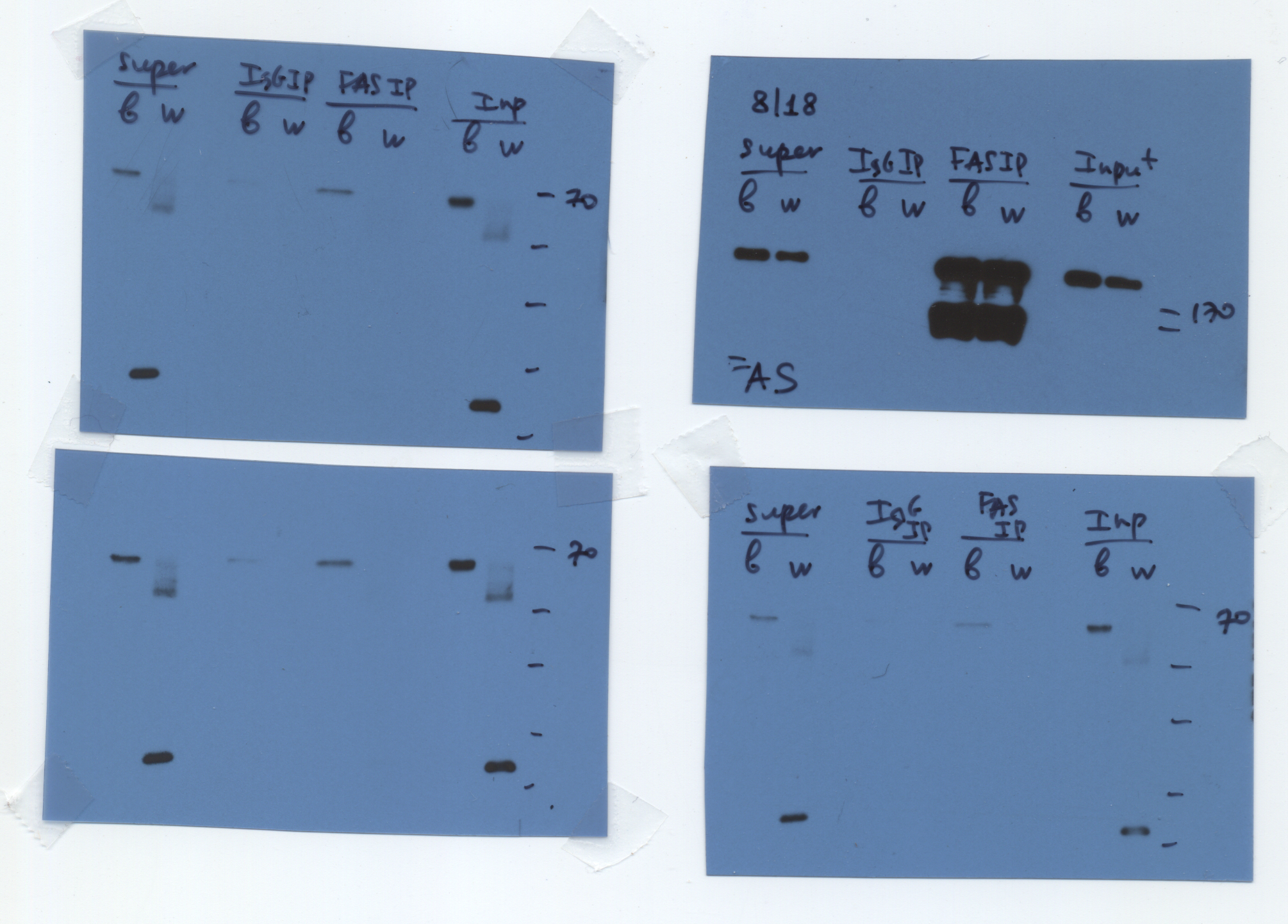

Supplement: Figure 4—source data 3. [file elife-67358-fig4-data3.zip › ddbceeb3-2ddd-4afc-b2fe-c98ee6f89ec2.jpeg]

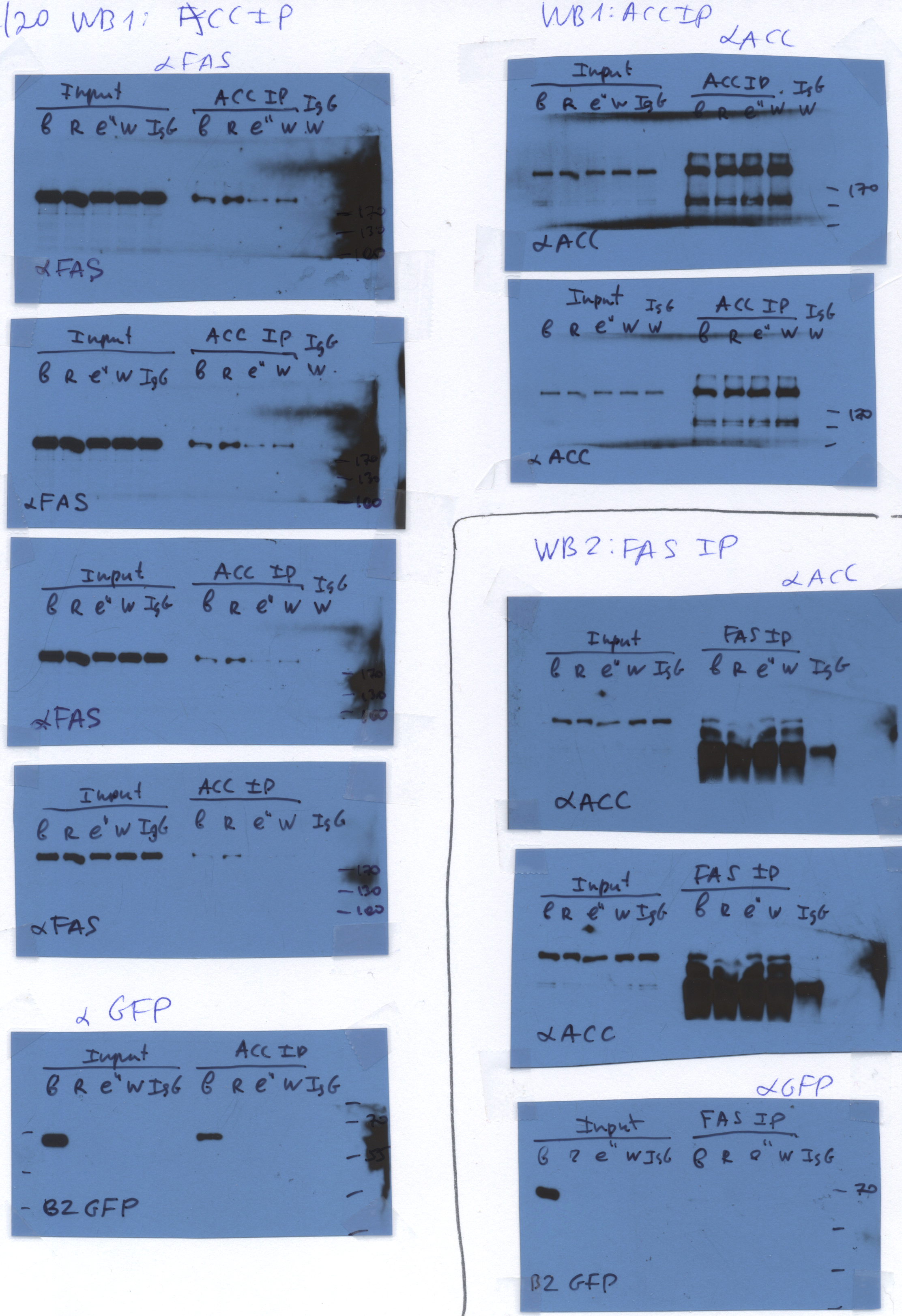

Supplement: Figure 4—source data 4. [file elife-67358-fig4-data4.zip › 560d09ed-503c-4444-8a1a-10012d3a2b46.jpeg]

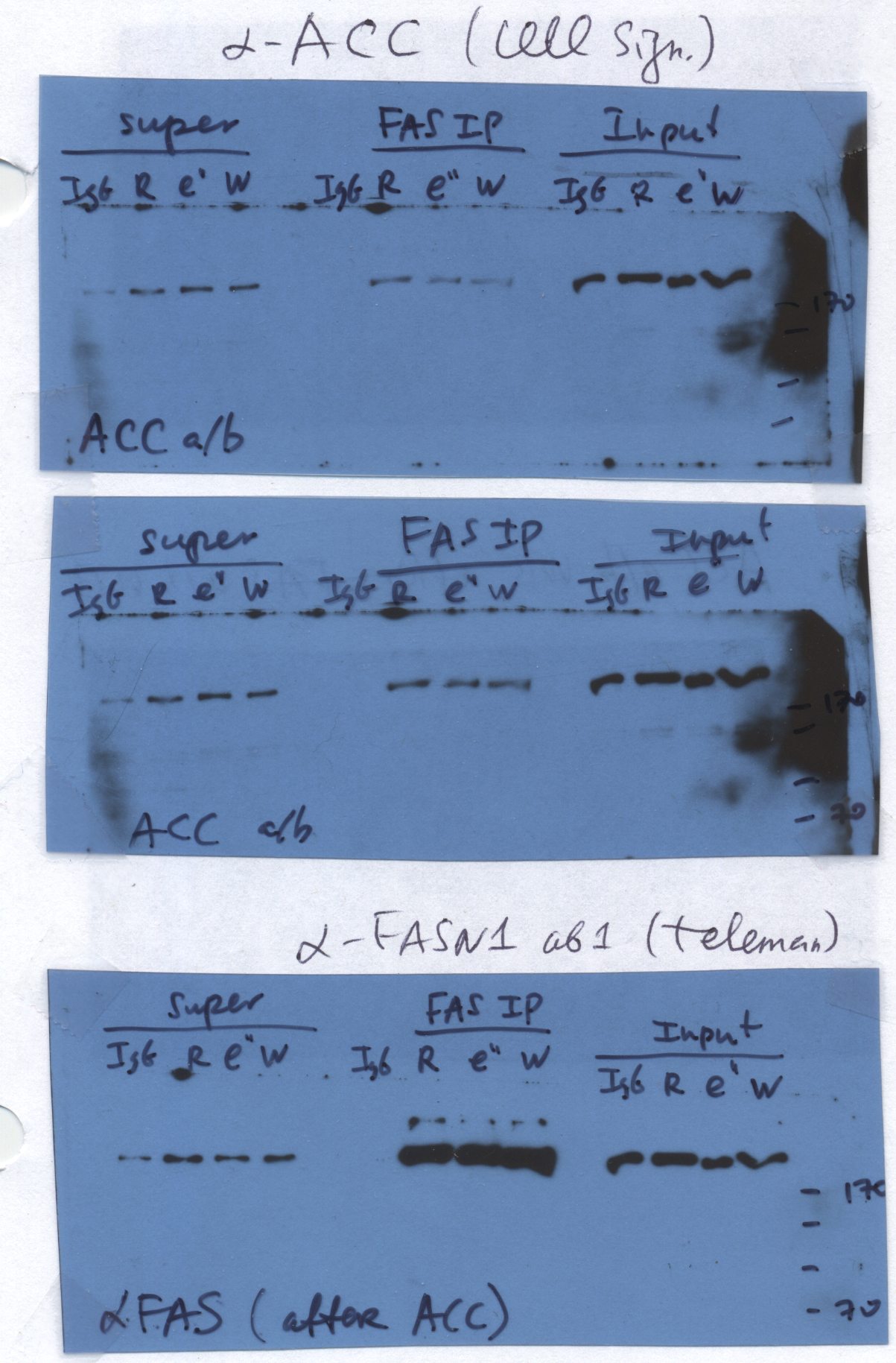

Supplement: Figure 4—source data 5. [file elife-67358-fig4-data5.zip › dd942663-835b-47c4-a3a7-7a8678b8c6e5.jpeg]

Source data for Figure 5A'

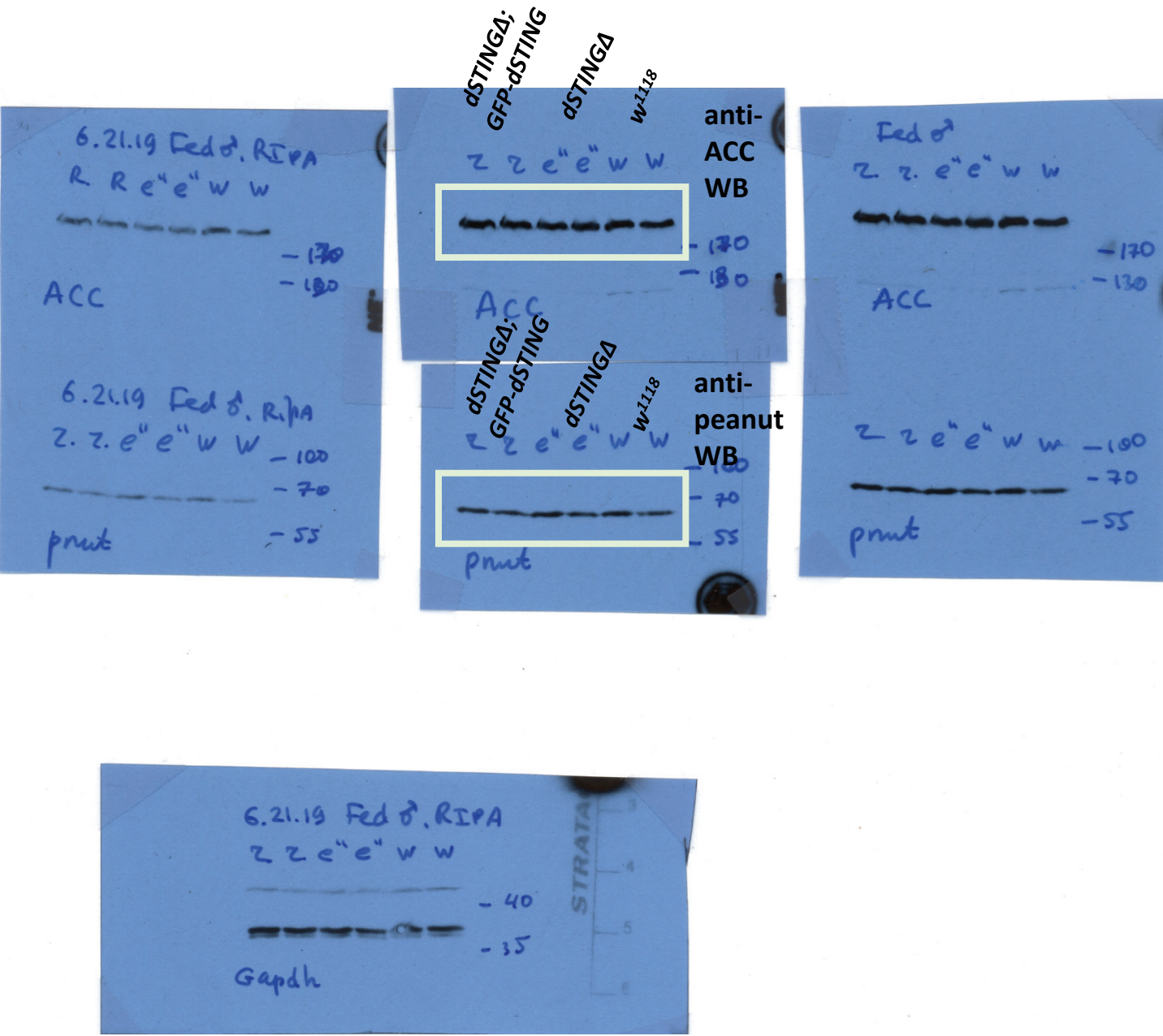

Source data for Figure 5B'

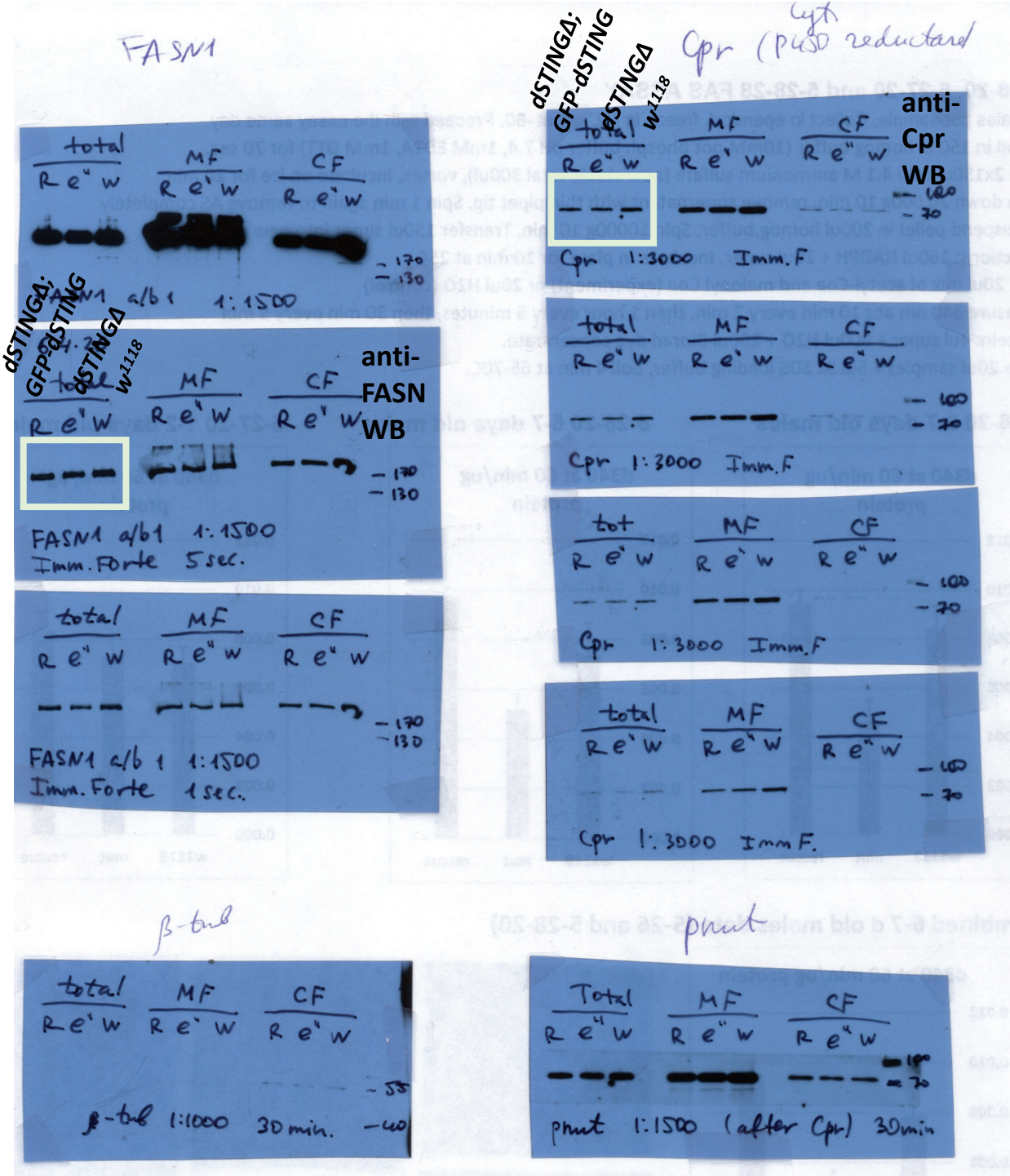

Supplement: Figure 5—source data 1. [file elife-67358-fig5-data1.pdf]

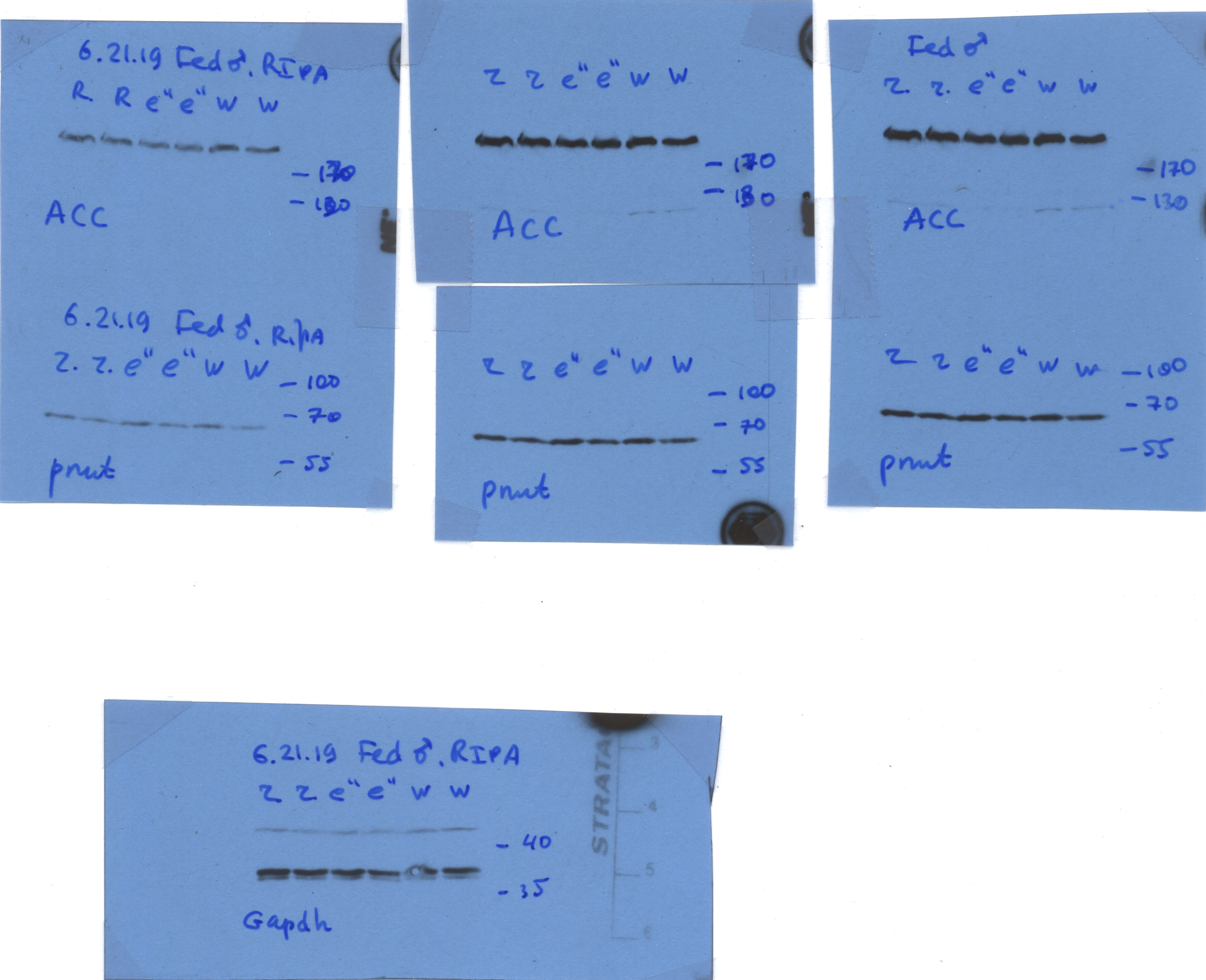

Supplement: Figure 5—source data 2. [file elife-67358-fig5-data2.zip › 6e2bcadc-340b-4cc2-95fd-1f624cc8f0dd.jpeg]

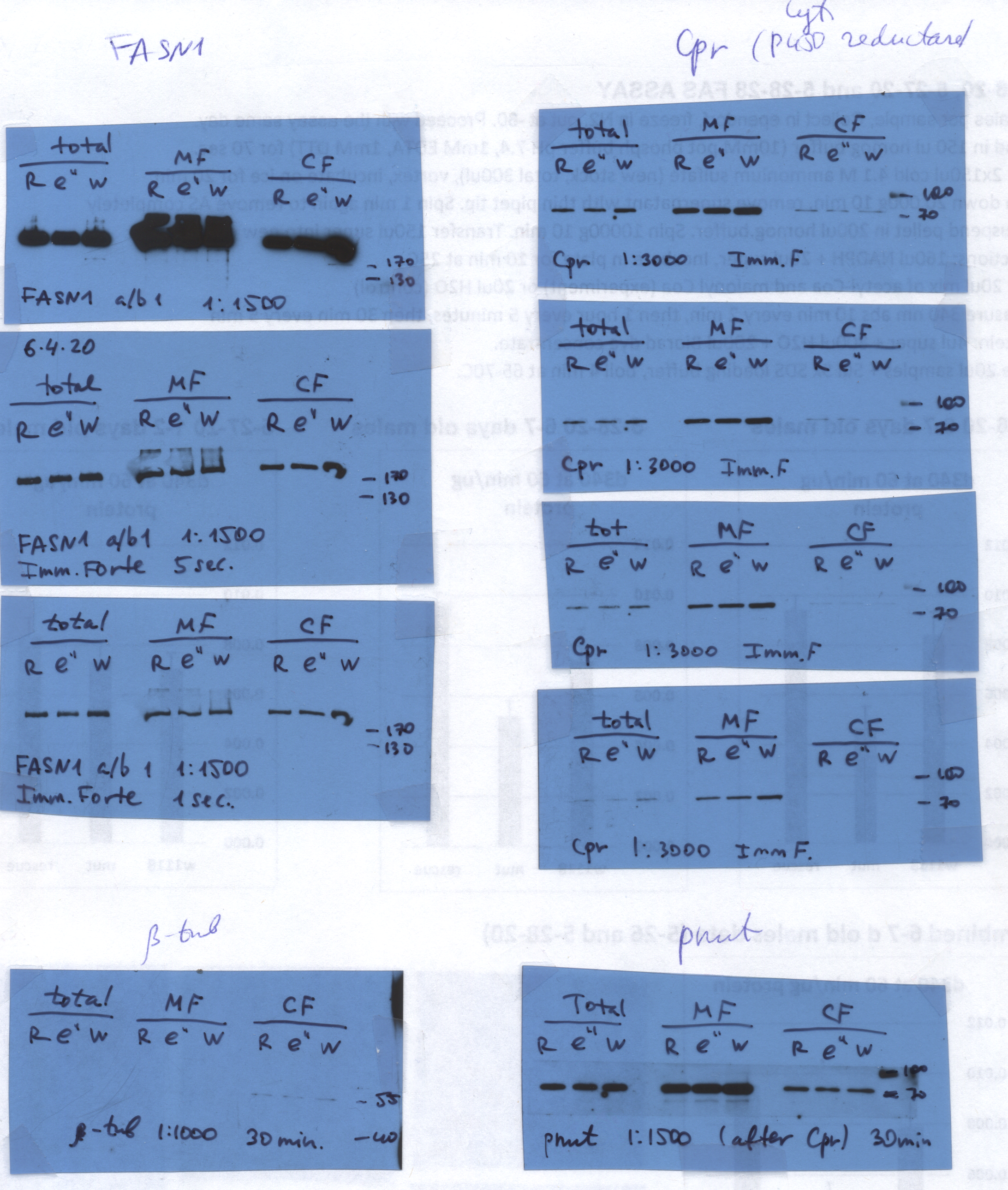

Supplement: Figure 5—source data 3. [file elife-67358-fig5-data3.zip › d8eaaa3f-b1eb-444a-aaa5-5c36abed40d4.jpeg]

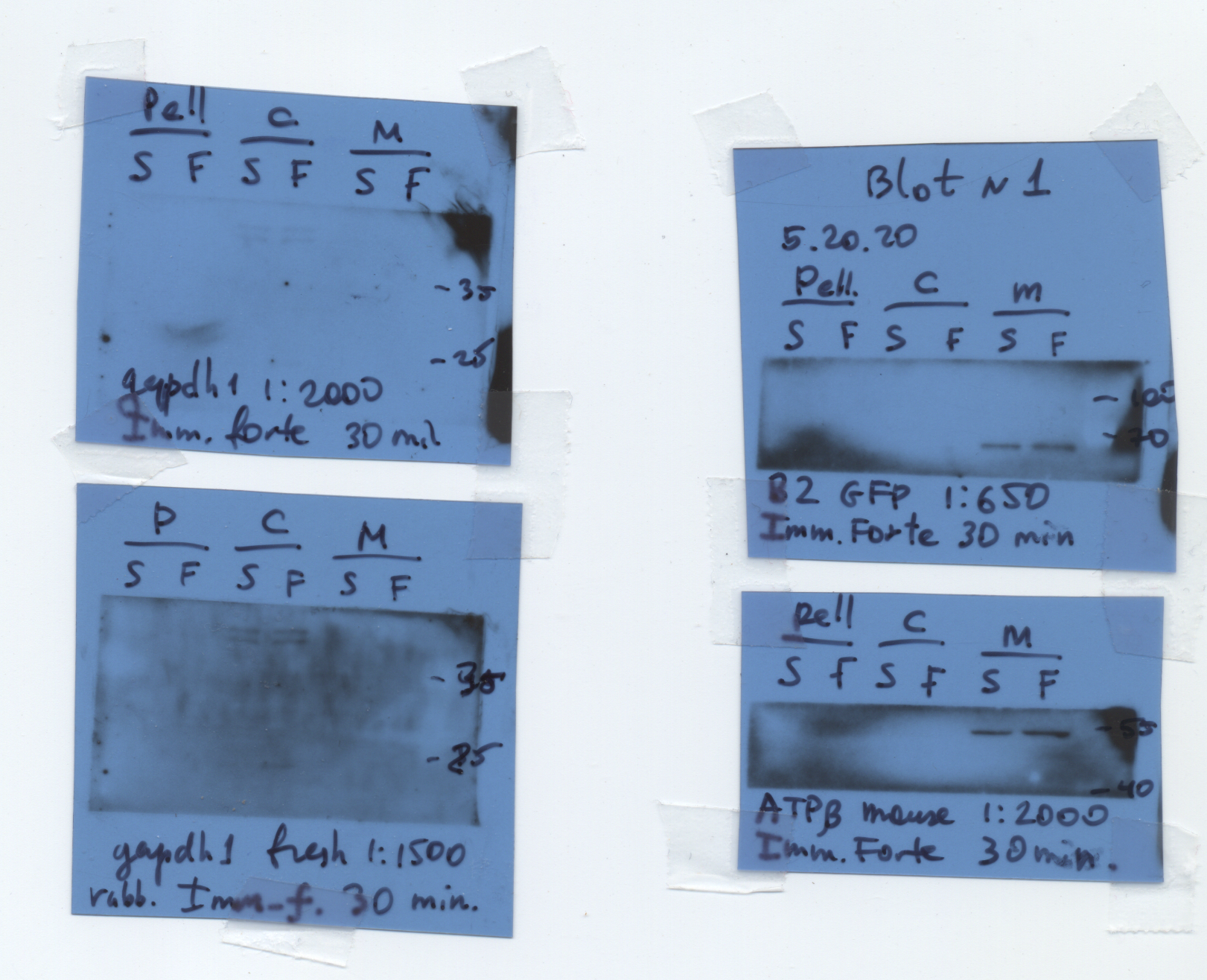

Supplement: Figure 6—figure supplement 1—source data 2. [file elife-67358-fig6-figsupp1-data2.zip › d3f52751-e0b8-4516-821c-16e60968ab7b.jpeg]
