# Supplementary material for: Drosophila STING protein has a role in lipid metabolism
Source: eLife. 2021 Sep 1;10:e67358. doi: 10.7554/eLife.67358 (PMC8443252; doi:10.7554/eLife.67358)

Source data for Figure 4A

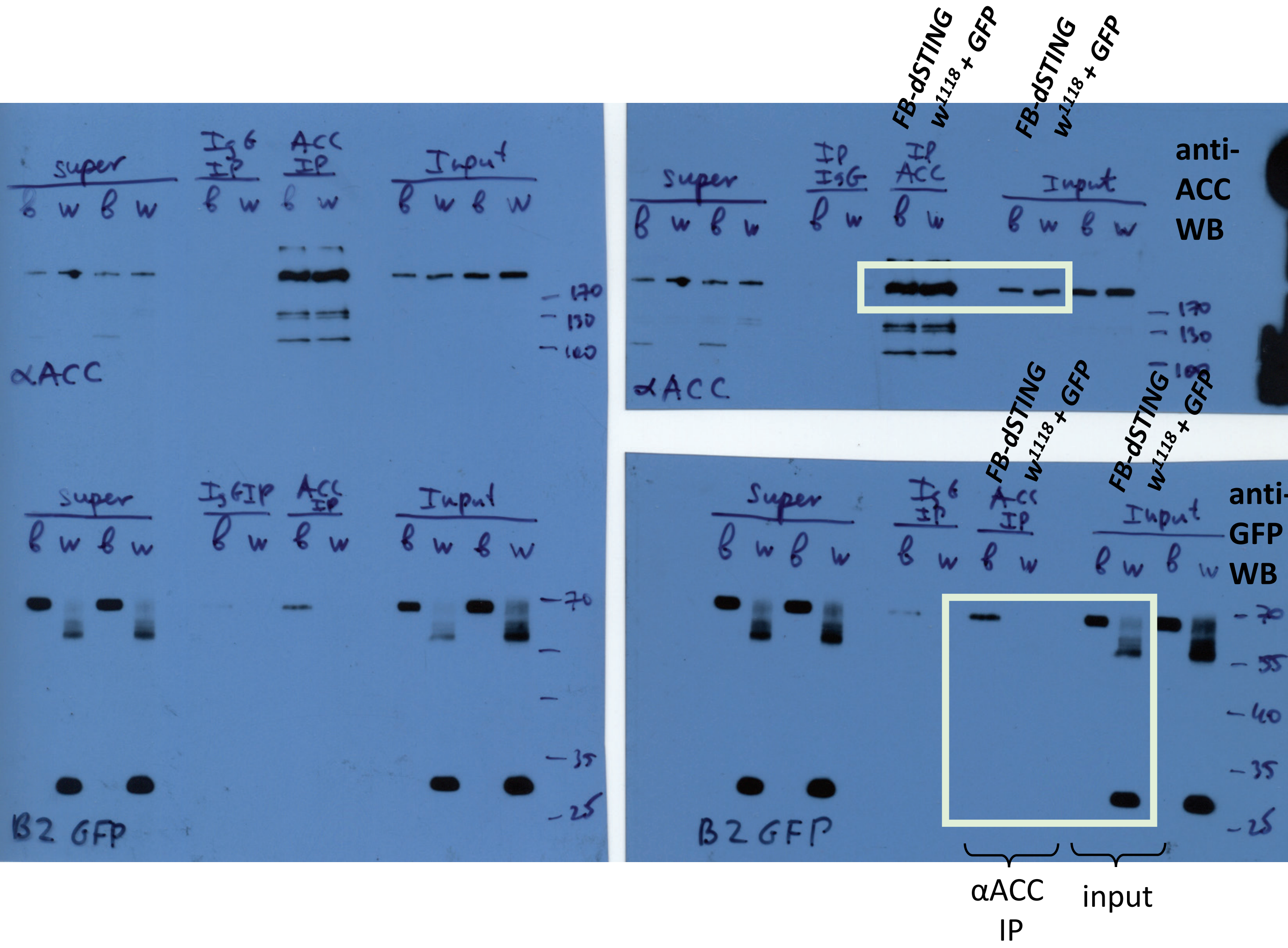

Source data for Figure 4B

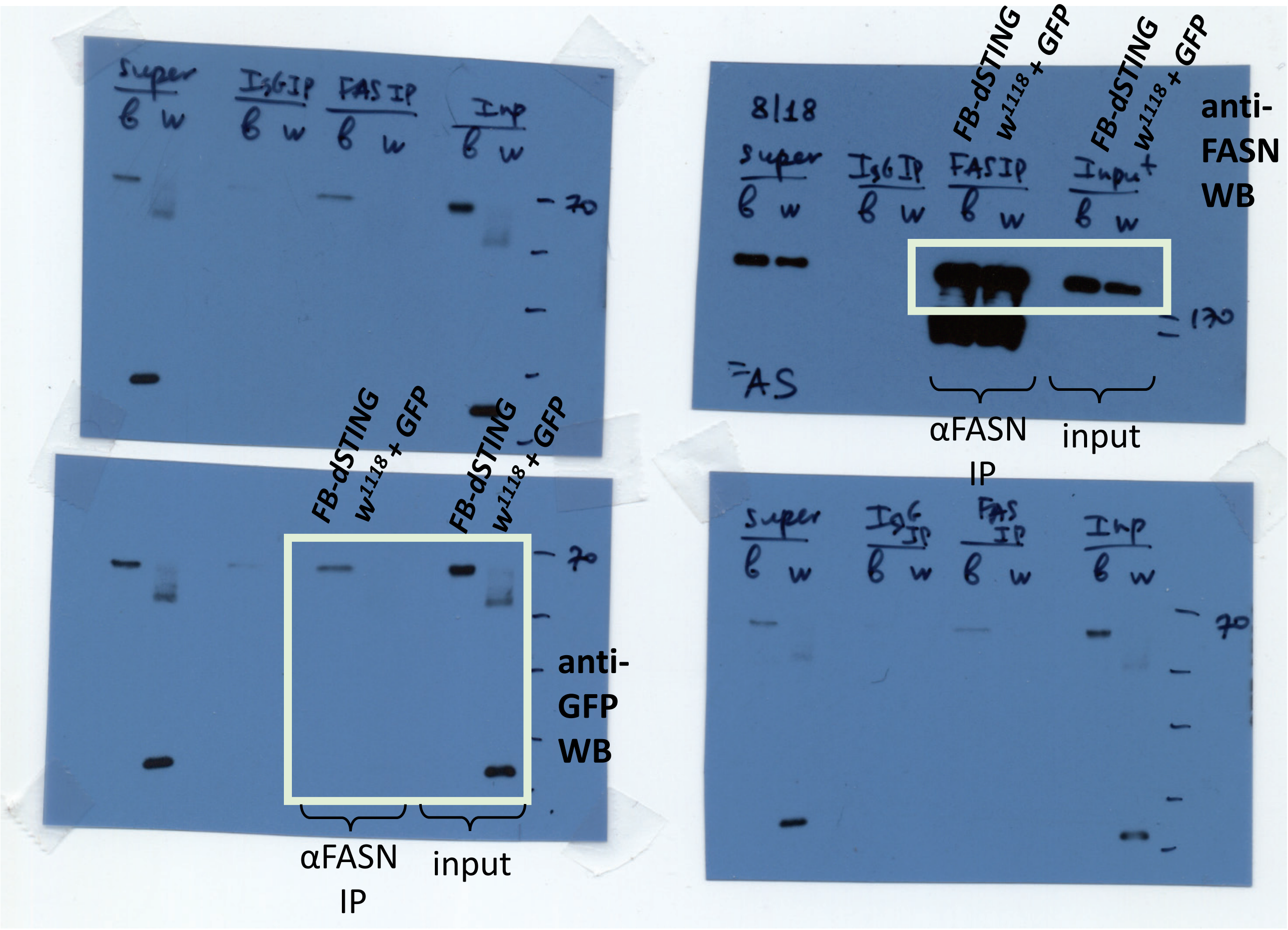

Source data for Figure 4C

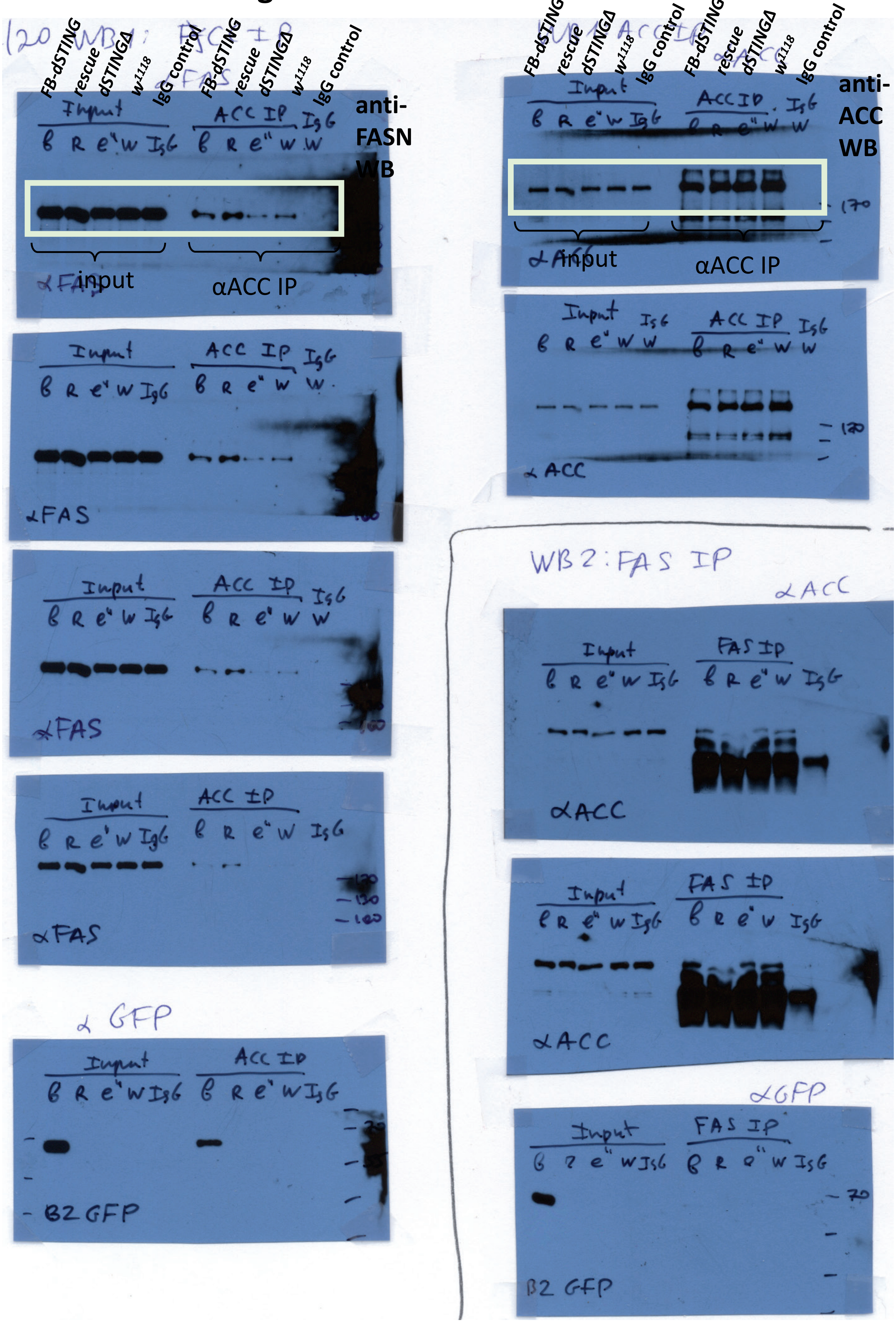

Source data for Figure 4D

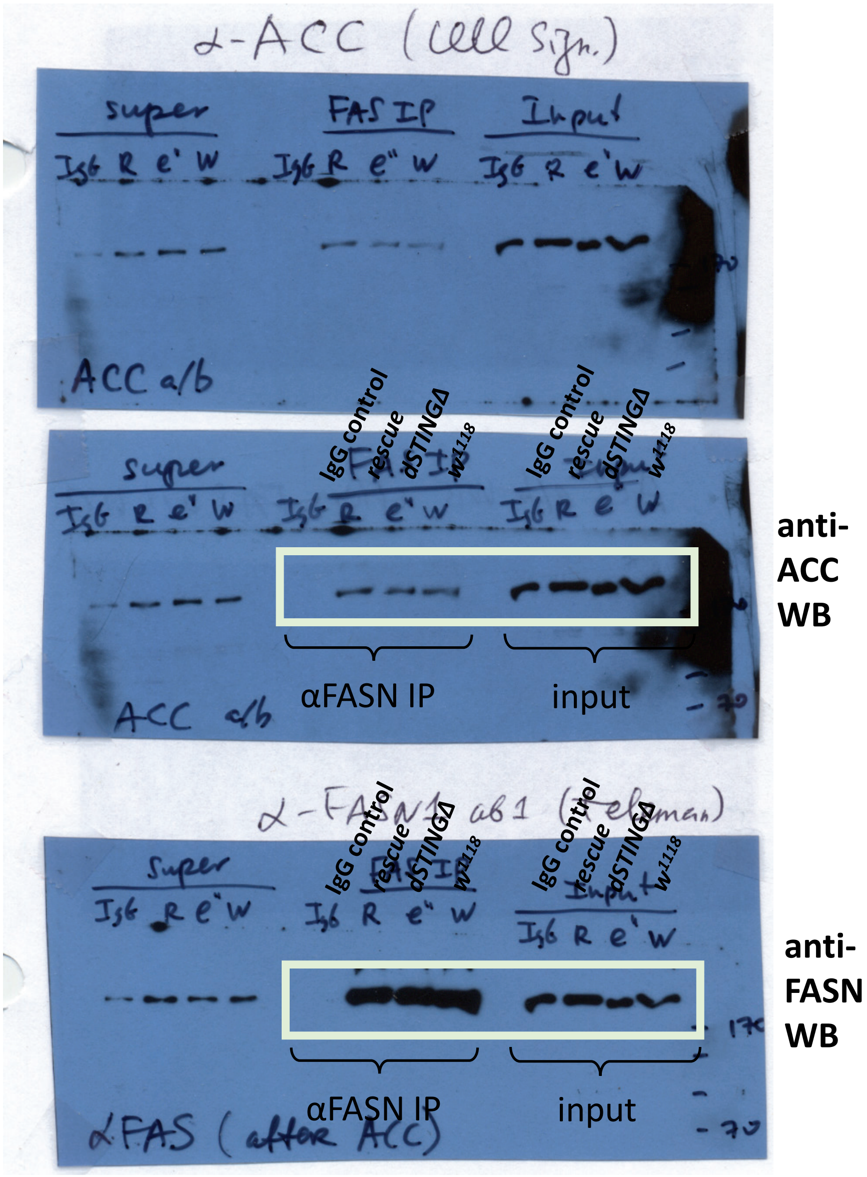

Supplement: Figure 4—source data 1. [file elife-67358-fig4-data1.pdf]
